# Single-stage outpatient laparoendoscopic treatment of cholecystocholedocholithiasis

**DOI:** 10.1590/0102-672020260000029e1958

**Published:** 2026-08-31

**Authors:** Gabriel Garcia SCHMITT, Alberto Facury GASPAR, Jorge Resende LOPES, Víctor Antônio Peres Alves Ferreira AVEZUM, Leonardo Santos LIMA, Rafael KEMP, Ajith Kumar SANKARANKUTTY, José Sebastião DOS SANTOS

**Affiliations:** 1 Universidade de São Paulo, Ribeirão Preto Faculty of Medicine, Department of Surgery and Anatomy – Ribeirão Preto (SP), Brazil.; 2 Universidade de São Paulo, Ribeirão Preto University Hospital – Ribeirão Preto (SP), Brazil.

**Keywords:** Cholelithiasis, Choledocholithiasis, Endoscopy, Ambulatory surgical procedures, Laparoscopy, Colelitíase, Coledocolitíase, Endoscopia, Procedimentos cirúrgicos ambulatórios, Laparoscopia

## Abstract

**Background:**

Single-stage outpatient treatment of cholecystocholedocholithiasis is feasible, highlighting the importance of appropriate patient selection, professional training, and healthcare service organization to support this approach.

**Aim:**

To identify clinical and procedural factors associated with outpatient management and hospital stay following single-stage laparoendoscopic treatment of cholecystocholedocholithiasis.

**Methods:**

A retrospective cohort study was conducted at hospitals affiliated with the Ribeirão Preto School of Medicine, Universidade de São Paulo (FMRP-USP), between 2019 and 2024. Patients were stratified into three groups according to care setting: outpatient (G1); outpatient with overnight stay (G2); and inpatient (G3). Clinical, surgical, and outcome data were analyzed using logistic regression models, χ^2^ tests, and Fisher’s exact tests (p=0.05).

**Results:**

Among 177 patients included, 41 were allocated to G1, 80 to G2, and 56 to G3. Compared with G2, G1 patients had shorter operative time (124.2 vs. 143.8 min; p=0.038), more frequent use of the Rendez-Vous technique (p=0.044), and less frequent use of papillary dilation (p=0.041). Patients in G3 had a higher prevalence of ASA III physical status (p=0.045), higher rates of postoperative complications (p=0.025) and biliary stent use (p=0.001), and lower bile duct clearance rates (p=0.004).

**Conclusions:**

Single-stage outpatient laparoendoscopic treatment of cholecystocholedocholithiasis is safe and effective. Clinical severity, treatment complexity, and failure of bile duct clearance were more strongly associated with inpatient hospitalization, whereas overnight stay among outpatients was not associated with improved postoperative outcomes. The implementation of enhanced recovery protocols and telemedicine-based postoperative assessment strategies may further reduce hospital stay.

## ARTICLE HIGHLIGHTS

Single-stage outpatient laparoendoscopic treatment of cholecystocholedocholithiasis is safe and effective.

Outpatient management of cholecystocholedocholithiasis without overnight stay is feasible in selected patients.

Clinical and surgical complexity and treatment failure are more strongly associated with inpatient hospitalization.

Multidisciplinary training, healthcare service organization, and optimization of postoperative care may contribute to reducing hospital stay.

## Summary

**CENTRAL MESSAGE:**

Single-stage laparoendoscopic treatment of cholecystocholedocholithiasis can be safely performed on an outpatient basis, including discharge without overnight hospitalization in selected patients. Clinical severity, treatment complexity, and failure of bile duct clearance were more strongly associated with inpatient hospitalization, whereas overnight stay among outpatients was not associated with improved postoperative outcomes. These findings support the feasibility of expanding ambulatory management strategies for cholecystocholedocholithiasis.

**PERSPECTIVES:**

The broader adoption of outpatient single-stage management of cholecystocholedocholithiasis requires advances in three key areas: multidisciplinary training, healthcare service organization, and perioperative care optimization. Enhanced Recovery After Surgery (ERAS) protocols and telemedicine-based postoperative assessment may facilitate earlier discharge while maintaining patient safety. Future prospective multicenter studies should validate patient-selection criteria, evaluate patient-reported outcomes and quality of life, and assess the economic impact of ambulatory management. Such evidence may support the development of standardized outpatient biliary surgery pathways and contribute to improving access, efficiency, and value in surgical care.

## VISUAL ABSTRACT

**Figure:**
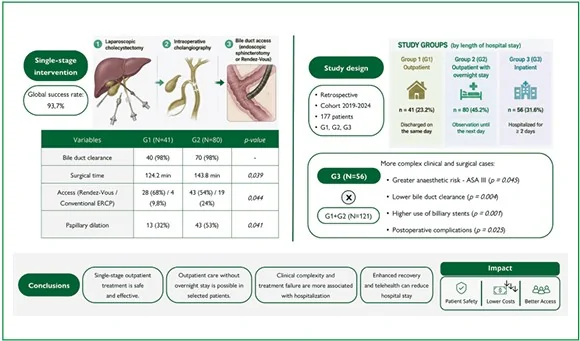

## RESUMO

**Racional:**

O tratamento da colecistocoledocolitíase pode ser feito em tempo único e em regime ambulatorial, o que justifica a seleção dos pacientes, a formação profissional e a organização dos serviços para garantir essa abordagem.

**Objetivos:**

Identificar os pacientes elegíveis ao tratamento da colecistocoledocolitíase em tempo único, em regime ambulatorial, e sem pernoite hospitalar.

**Métodos:**

Estudo de coorte retrospectivo nos hospitais vinculados à FMRP-USP entre 2019 e 2024. Os pacientes foram alocados em três grupos conforme o regime assistencial: G1) ambulatorial; G2) ambulatorial com pernoite hospitalar; e G3) internação. Os dados clínicos, cirúrgicos e de desfecho foram analisados por meio de modelos de regressão logística, testes χ^2^ e Fisher (p=0,05).

**Resultados:**

Dos 177 pacientes incluídos, 41 foram alocados no G1, 80 no G2 e 56 no G3. O G1 apresentou menor tempo cirúrgico (124,2 vs 143,8 min; p=0,038), maior emprego da técnica Rendez-Vous (p=0,044) e menor uso da dilatação papilar (p=0,041) em relação ao G2. No G3, os pacientes apresentaram maior risco anestésico ASA III (p=0,045), maior taxa de complicações pós-operatórias (p=0,025), maior emprego de prótese biliar (p=0,001) e menor taxa de clareamento biliar (p=0,004).

**Conclusões:**

O tratamento laparoendoscópico da colecistocoledocolitíase em tempo único e em regime ambulatorial é seguro e eficaz. A maior gravidade clínica, a maior complexidade terapêutica e a falha no clareamento da via biliar estiveram associadas à necessidade de internação, enquanto o pernoite hospitalar entre pacientes tratados em regime ambulatorial não se associou a melhores desfechos pós-operatórios. A implementação de protocolos de Recuperação Rápida após Cirurgia (ERAS) e de estratégias de teleavaliação pós-operatória pode contribuir para reduzir ainda mais a permanência hospitalar.

## INTRODUCTION

 Cholecystocholedocholithiasis is present in approximately 10–20% of patients with symptomatic gallstone disease^7,12^ and is associated with severe conditions such as cholangitis and pancreatitis, which increase diagnostic and therapeutic complexity, morbidity, mortality, and overall healthcare costs^15^. 

 Depending on its severity, cholangitis may require emergency management; however, treatment is preferably performed in an urgent elective setting^28^. 

 Single-stage treatment of cholecystocholedocholithiasis may be performed either through a fully laparoscopic approach combining laparoscopic cholecystectomy with bile duct exploration or through a laparoendoscopic approach, in which endoscopic stone extraction and cholecystectomy are performed during the same surgical procedure. These strategies have demonstrated favorable outcomes, improved cost-effectiveness, and lower complication rates^13,20,29^. 

 The laparoscopic approach offers the advantages of minimally invasive surgery while reducing the number of procedures per patient, shortening hospital stay, and avoiding manipulation of the sphincter of Oddi, thereby eliminating risks associated with endoscopic sphincterotomy^4,5,18^. Nevertheless, the laparoendoscopic approach has emerged as an attractive minimally invasive alternative as it avoids choledochotomy and abdominal drain placement while demonstrating favorable cost-effectiveness, particularly when performed using the Rendez-Vous technique^10,19,21^. 

 Traditionally, these single-stage procedures have been performed during inpatient hospitalization. However, our institution has pioneered their implementation in an outpatient setting, including in medium-complexity hospitals, with satisfactory safety and cost-reduction outcomes, particularly during the COVID-19 pandemic^8,17^. This experience highlights the potential for restructuring educational and healthcare delivery processes, especially through multidisciplinary training, optimization of perioperative workflows, and implementation of enhanced recovery and telemedicine-based postoperative follow-up protocols. 

 The present study aimed to identify clinical and procedural factors associated with outpatient management and hospital stay following single-stage laparoendoscopic treatment of cholecystocholedocholithiasis. 

## METHODS

 This retrospective cohort study included all patients with suspected cholecystocholedocholithiasis and a confirmed diagnosis who underwent single-stage laparoendoscopic treatment from January 2019 to December 2024 at two public teaching hospitals affiliated with the Ribeirão Preto School of Medicine, Universidade de São Paulo (FMRP-USP), Hospital das Clínicas de Ribeirão Preto (HCRP), a tertiary-level referral center for hepatobiliopancreatic surgery and endoscopy, and Hospital Estadual de Ribeirão Preto (HERP), a medium-complexity institution without an intensive care unit. 

 The study was conducted in accordance with the guidelines of the institutions’ Research Ethics Committee, under approval number 7.030.384, and was registered on Plataforma Brasil under CAAE 79894624.1.0000.5440. 

 All patients first underwent laparoscopic cholecystectomy associated with intraoperative cholangiography for confirmation of choledocholithiasis. Bile duct access was achieved during the same procedure using either endoscopic retrograde cholangiopancreatography (ECRP) with endoscopic sphincterotomy or the Rendez-Vous technique through anterograde cystic duct cannulation and guidewire externalization via the duodenal papilla. The bile duct was subsequently swept for stone extraction, with or without duodenal papilla dilation, followed by contrast injection to confirm bile duct clearance. All procedures were performed by the same surgical team (Video: https://youtu.be/COZxwgfA4Bo). 

 Demographic and clinical variables (age, gender, anesthetic risk, and care setting), surgical variables (bile duct access technique, papillary dilation, biliary stent placement, and operative time), and postoperative outcomes (treatment success, complications, abdominal drain use, and readmissions) were collected. 

### Inclusion and exclusion criteria

 Patients older than 17 years with a diagnosis of cholecystocholedocholithiasis confirmed by intraoperative cholangiography were considered eligible for inclusion in the study. The following patients were excluded: those with negative intraoperative cholangiography findings; severe cholangitis or acute cholecystitis according to the 2018 Tokyo criteria^28^; moderate to severe pancreatitis according to the 2012 Atlanta criteria^6^; a definitive diagnosis or suspicion of other hepatobiliopancreatic conditions, such as neoplasms, Mirizzi syndrome, primary sclerosing cholangitis, or cirrhosis; previous upper gastrointestinal tract anastomoses; contraindications to general anesthesia and pneumoperitoneum; and American Society of Anesthesiologists (ASA) physical status classes IV, V, and VI^3^. 

### Statistical analysis

 All analyses were performed using R statistical software, version 4.3.2. Statistical significance was set at p<0.05. Descriptive analyses were conducted using measures of central tendency and dispersion (mean, standard deviation, and median) for continuous variables and frequencies and percentages for categorical variables. 

 For the binary outcome variable (group), logistic regression models were used to evaluate associated factors and identify the simplest model describing the relation between the binary dependent variable and a set of independent variables^16^. For analyses involving categorical variables, the χ^2^ test or Fisher’s exact test was used, according to the frequencies observed in the cohort. 

## RESULTS

**Figure 1:**
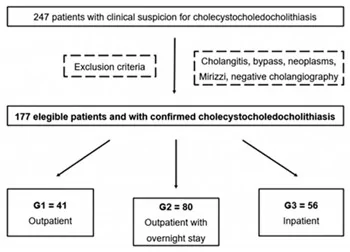
Patient selection and distribution among groups (G1, G2, and G3).

 A total of 177 patients were included in the cohort (Figure 1):

- G1: 41 outpatients;
- G2: 80 outpatients with overnight stay;
- G3: 56 inpatients.

 Most patients were female (70.6%), with a mean age of 49.3 years, and most procedures were performed at the University Hospital of Ribeirão Preto. Notably, during the COVID-19 pandemic, 28 patients (16%) underwent treatment at HERP. The overall bile duct clearance rate was 93.8%, whereas postoperative complication and readmission rates were 18.6 and 5.6%, respectively. Patients in G3 showed lower treatment success rates and higher rates of clinical and surgical complications (Table 1). No deaths were recorded. 

**Table 1:** Analysis of groups profiles regarding age, gender, treatment center, complications, abdominal drain use, readmissions, and bile duct clearance.

Variable		G1 (n=41)	G2 (n=80)	G3 (n=56)	Total (N=177)
Age		46.95	49.65	50.53	49.27
Female, n (%)		30 (73.2)	56 (70.0)	39 (69.6)	125 (70.6)
Male, n (%)		11 (26.8)	24 (30.0)	17 (30.4)	52 (29.4)
Treatment					
	HCRP, n (%)	28 (68.3)	73 (91.3)	48 (85.7)	149 (84.2)
	HERP, n (%)	13 (31.7)	7 (8.8)	8 (14.3)	28 (15.8)
	Complications, n (%)	5 (12.2)	8 (10.0)	20 (35.7)	33 (18.6)
	Abdominal drain use, n (%)	2 (4.9)	10 (12.5)	15 (26.8)	27 (15.3)
	Readmissions, n (%)	2 (4.9)	5 (6.3)	3 (5.4)	10 (5.6)
	Bile duct clearance, n (%)	40 (97.6)	78 (97.5)	48 (85.7)	166 (93.8)

G1: outpatient; G2: outpatient with overnight stay; G3: inpatient; HCRP: Hospital das Clínicas de Ribeirão Preto; HERP: Hospital Estadual de Ribeirão Preto.

 The clinical profiles of patients in G1 and G2 were similar, and the main differences were related to procedural and perioperative factors (Table 2). Compared with G2, G1 patients had shorter operative time (p=0.039), more frequent use of the Rendez-Vous technique than conventional ERCP (p=0.044, p<0.05), and less frequent use of duodenal papilla dilation (p=0.041, p<0.05). 

**Table 2:** Comparative analysis between G1 (outpatient) and G2 (outpatient with overnight stay) regarding clinical characteristics, surgical techniques, and outcomes.

Variable	G1 (n=41)	G2 (n=80)	p-value**
Age (years), mean	46.95	49.65	0.86
ASA II, n (%)	25 (61.0)	53 (66.3)	0.58
ASA III, n (%)	3 (7.3)	8 (10.0)	0.96
Access – Rendez-vous, n (%)	28 (68.3)	43 (53.8)	0.044†
Access – Conventional ERCP, n (%)	4 (9.8)	19 (23.8)	—
Papillary dilation*, n (%)	13 (31.7)	42 (52.5)	0.041†
Bile duct clearance, n (%)	40 (97.6)	78 (97.5)	0.35
Biliary stent, n (%)	0 (0.0)	2 (2.5)	0.54
Operative time (min), mean	124.54	143.83	0.039†
Complications, n (%)	5 (12.2)	8 (10.0)	0.94
Abdominal drain use, n (%)	2 (4.9)	10 (12.5)	0.46

†p<0.05; ASA: American Society of Anaesthesiologists Classification; ERCP: endoscopic cholangiopancreatography; *χ^2^ test; **logistic regression model.

 When the outpatient groups (G1+G2) were compared with hospitalized patients (G3), ASA III status was more frequent in G3 (p=0.04, p<0.05). In addition, G3 showed lower bile duct clearance rates (p<0.01), greater use of biliary stents (p<0.01), and higher rates of postoperative complications (Table 3). 

**Table 3:** Comparative analysis between outpatient (G1 + G2) and inpatient (G3) groups regarding age, anesthetic risk, surgical techniques, bile duct clearance, and outcomes.

Variable	G1 + G2 (n=121)	G3 (n=56)	p-value**
Age (years), mean	48.30	50.43	0.27
ASA II, n (%)	78 (64.5)	33 (58.9)	0.33
ASA III, n (%)	11 (9.1)	11 (19.6)	0.04†
Papillary dilation, n (%)	55 (45.5)	30 (53.6)	0.53
Biliary stent*, n (%)	2 (1.7)	8 (14.3)	<0.01†
Bile duct clearance*, n (%)	118 (97.5)	48 (85.7)	<0.01†
Operative time (min), mean	137.40	156.95	0.30
Abdominal drain use, n (%)	12 (9.9)	15 (26.8)	0.36
Complications, n (%)	13 (10.7)	20 (35.7)	0.02†

†p<0.05; ASA: American Society of Anaesthesiologists Classification; *Fisher’s exact test; **logistic regression model.

## DISCUSSION

 Several therapeutic algorithms have been proposed for the management of cholecystocholedocholithiasis^5,18^; however, single-stage laparoendoscopic treatment has consistently demonstrated favorable safety and cost-effectiveness outcomes, particularly when performed using the Rendez-Vous techniques^10,20^. Recent observational studies and clinical trials have also demonstrated the success of this approach in an outpatient setting, which motivated the present investigation^8,17^. 

 In the current study, higher anesthetic-surgical risk, increased clinical complications, and failure of bile duct clearance were associated with the need for inpatient hospitalization and were predominantly observed in G3. In contrast, comparison between G1 and G2 revealed mainly differences in procedural complexity, without clinically significant postoperative outcomes that justified overnight hospitalization. Therefore, strategies aimed at reducing unnecessary overnight stays should be considered. 

 Implementation of single-stage outpatient management of cholecystocholedocholithiasis requires structural modifications involving three major domains: multidisciplinary training, healthcare service organization, and optimization of postoperative care. 

 Laparoendoscopic surgery requires not only mastery of two access methods for the treatment of cholecystocholedocholithiasis but also a complex logistical framework for integrating these methods. This study shows that surgeon training focused on patient needs, expanding access to treatment, and improving cost-effectiveness is already a reality in public teaching hospitals in Brazil; however, this paradigm shift faces challenges that are not only practical but also institutional, regarding the training and practice of surgeons and endoscopists. 

 The organization of healthcare services also plays a central role in enabling ambulatory management, since hospital stay is often more closely related to institutional workflow than to clinical criteria alone. Factors such as operating room turnover, anesthetic recovery time, shift transitions, and delays in postoperative evaluation may unnecessarily prolong hospitalization and increase costs^2,9^. Optimizing these factors through operational protocols can help facilitate outpatient surgeries, as has occurred with laparoscopic cholecystectomy in recent years, which is now performed routinely in medium-complexity public hospitals^22,25^. 

 Enhanced recovery after surgery (ERAS) protocols have demonstrated efficacy in reducing postoperative morbidity and hospital stay, particularly in digestive surgery, through strategies including multimodal analgesia, optimization of comorbidities, reduced fasting periods, and early mobilization^1,11,23^. However, communication gaps among teams, lack of personalized care, and even surgeons’ resistance to implementing new protocols are factors that hinder its systematic adoption^26^. 

 In addition, telemedicine-based postoperative follow-up may further facilitate early discharge by improving adherence to postoperative care and enabling early identification of complications, prevention of readmissions, and, most importantly, reduction in hospital stay^24,27^. Features such as video calls with the medical team and mobile health applications have already been associated with increased adherence and positive feedback^14^. Thus, tele-assessment may be a viable option for patient follow-up and guidance, with potential application in the treatment of cholecystocholedocholithiasis to reduce hospital stay. 

 In the present study, this discussion is particularly relevant considering the potential for modifying the care setting for G2, since, given the similarity in clinical profiles and outcomes, these patients could have received the same treatment as G1, thereby avoiding overnight hospitalization and unnecessary costs. Thus, it is considered that the results presented, although unprecedented, may be further improved through the implementation of more modern care and educational processes, primarily aimed at providing safe treatment with greater access and better cost-effectiveness for patients and health systems. 

## CONCLUSIONS

 The findings of this study suggest that outpatient single-stage management of cholecystocholedocholithiasis can be safely expanded in selected patients. Clinical severity, treatment complexity, and failure of bile duct clearance were more strongly associated with inpatient hospitalization than with the need for overnight stay among outpatients. The adoption of enhanced recovery protocols and telemedicine-based postoperative assessment may further reduce hospital stay, while future prospective multicenter studies should define objective patient-selection criteria and evaluate the clinical and economic impact of this treatment strategy. 

## Data Availability

The datasets generated and/or analyzed during the current study are available from the corresponding author upon reasonable request. The information regarding the investigation, methodology and data analysis of the article is archived under the responsibility of the authors.
